# Clinical Lectures on Surgery, Delivered at St. George’s Hospital

**Published:** 1846-05

**Authors:** 


ART. VII.—*Clinical Lectures on Surgery, delivered at St. George's Hospital.* By SIR BENJAMIN C. BRODIE, Bart. V.P.R.S.; Sergeant Surgeon to the Queen; Surgeon in Ordinary to his Royal Highness Prince Albert, &c., &c., &c. Philadelphia: Lea & Blanchard. 1846. 8 vo. pp. 352.

The introductory lecture to the course of lectures comprised in this volume predisposes one strongly in favor of the volume and of its author. It breathes so kindly a spirit, manifests so lively an interest in the inexperienced young student, cast among strangers in a populous city, and points out with so much good sense the course of study and behavior to be observed, that the reader sets out fully certified of the benevolence of the author's heart, as well as of the soundness of his head. Sir Benjamin Brodie's is a name familiar to the American profession, and his clinical lectures, now made available, will increase his popularity on this side of the Atlantic. They will be found to be characterized, in a striking degree, by clearness of language, good sense, and a practical bearing. The course embraces thirty-nine lectures in which the following subjects are discussed: Some of the important circumstances connected with operative surgery; mortification; inflammation of the veins; varicose veins and ulcers of the legs; corns and bunions; polypi of the nose; non-malignant tumors of the tongue; paralysis; extraction of foreign bodies; fistula in ano; fatty steatomatous tumors; sero-cystic tumors of the breast; scirrhus of the breast; the administration of mercury in syphilis; local nervous affections; various forms of local hysterical affections; diseases of the hip joint; tic douloureux; hemorrhoids; prolapsus and excrescences of the rectum; preternatural contraction of the sphincter ani; ulcer on the inside of the rectum; stricture of the rectum; malignant diseases of the rectum; diseases of the maxillary antrum, and encysted tumors.

Two lectures are taken up with illustrations of some of the important circumstances connected with operations in surgery, and they are lectures which will be read with great pleasure. The illustrations are numerous and apposite, furnished by the large experience of the author. He begins by referring to the fact, that, while the efficacy of our remedies in most cases of disease may be a question, in surgical operations the success or failure must be a matter of certainty. If a cure arise from an operation it is attributed to that, and that only; and if a false step be taken it is apparent to the bystanders, and brings reproach upon the surgeon. We remember well a case in point, where a surgeon of our acquaintance performed the operation of lithotomy, and when he had got into the bladder found no stone there. It is true he was able to refer to Dupuytren, as having been betrayed into a similar error; but this was not sufficient to avert from his own head the disgrace of such a blunder. The examples mentioned by our author in the subjoined quotation place the subject in a strong light:

"But an operation, while it may do good, may also be productive of evil. A man has a stone in the bladder; he is suffering torture; he has nothing but a frightful death to which he can look forward. As the least of two evils, he is content to submit to the operation of lithotomy: and, it may be, that in the brief space of three minutes he is placed in a situation of perfect comfort, and that in forty-eight hours you are able to declare with confidence that his life is perfectly safe. A man may have a disease of the knee-joint, with carious bone and abscesses; he may be worn out by pain, by perspirations, sleepless nights, and other symptoms of hectic fever. You amputate the limb; and even on that very night he may sleep soundly; there may be no more perspirations; and in a week he may be gaining flesh, and present the aspect of health. But then, on the other hand, there are other cases, in which the patient, after lithotomy, may die within forty-eight hours, although he might have lived—in misery, it is true—had he been let alone, for a year or longer. So, in the case of amputation for a diseased knee-joint, the patient, instead of recovering, may die in the course of a few days, and very much sooner than he would have done had not an operation been resorted to."

An accomplished operator, Sir Benjamin Brodie considers a man who looks well at all the consequences of an operation before he begins it, and then thinks only of the patient, and not of the figure he may be making with the bystanders.—Never, he says, "slur over a single case;" but before going about the operation consider well all the accidents that may occur. He also urges the propriety of communicating to the patient or his friends your estimate of the dangers attending the operation. And, very prudently, he cautions operators against the error of making light of trivial operations, remarking that fatal results have attended as trifling wounds as a bee sting or a leech bite.

Hemorrhage, the shock to the nervous system, erysipelatous, and other forms of inflammation, and tetanus are among the consequences to be dreaded after surgical operations, and all have often lead to fatal terminations. The judgment of the surgeon is evinced in foreseeing the danger of these occurrences, and taking steps to prevent them.

The subject of mortification is treated at great length, but we pass it over, together with a number of other topics, in order to give some account of the lecture devoted to the local nervous affections, which Sir Benjamin introduces with the following case:

"A middle aged lady, who had been exposed during a considerable period of time to the operation of causes of great mental anxiety, complained of a constant and severe pain, which she referred to a spot, about three or four inches in diameter, in the situation of the false ribs of the left side. Besides this she was subject to fits, apparently connected with hysteria, and was otherwise in a very impaired state of health. Under these circumstances she died, and on examining the body after death, particular attention was paid to the side to which the pain had been referred. No morbid appearances could be detected in it; there was neither inflammation, nor thickening nor adhesion, nor any morbid change of structure, nor the slightest deviation of any kind from the natural condition of the part."

Upon which he remarks as follows:

"Now such a case as this is by no means uncommon. It is only one of many which might be adduced in proof of this proposition, namely, that the natural sensations of a part may be increased, diminished, or otherwise perverted, although no disease exists in it which our senses are able to detect either before or after death."

Often, however, there is a sufficient cause for these local nervous affections, as the subjoined instructive case will testify:

"A man was admitted into St. George's Hospital in the year 1808, complaining of a severe pain in the inside of his knee. The joint was carefully examined, but no marks of disease could be detected in it. In the thigh, however, there was an aneurism of the femoral artery, of the size of a small orange. This last disease had scarcely attracted the patient's notice. He said that he should be very well if it were not for the pain in the knee, and it was not until some trouble had been taken to explain to him the nature of his case, that he could be made to understand that the tumor was of any importance. Soon after the man's admission, Sir Evarard Home (then Mr. Home) applied a ligature round the femoral artery, in the upper part of the thigh. On the instant of the artery being secured the tumor ceased to pulsate, and the pain in the knee ceased also. Some untoward circumstances occurred, and the patient died about four or five days after the operation was performed. On inspecting the limb after death, the aneurism was found reduced to one-half of its former size; some branches of the anterior crural nerve, which passed over it, and which must have been kept on the stretch previous to the operation, were found to terminate in the part to which the pain had been referred, on the inside of the knee; and thus the cause of the pain was sufficiently explained. It was, in fact, a nervous pain, existing where there was no disease, in consequence of pressure on the nerves above."

Many analogous cases are recited in these lectures which will arrest the attention of the reader. Every practitioner of experience must have met with cases similar to the following:

"A gentleman awoke in the middle of the night, laboring under a severe pain in one foot: at the same time that some other sensations to which he was not unaccustomed, indicated the existence of an unusual quantity of acid in the stomach. To relieve the latter he swallowed a large dose of an alkaline medicine. Immediately on the acid in the stomach having been thus neutralized, the pain in the foot left him.

"The late Dr. Wollaston was accustomed to relate the following history:—He ate some ice-cream after dinner, which his stomach seemed to be incapable of digesting. Sometime afterwards, when he had left the dinner-table to go to the drawing-room, he found himself lame from a violent pain in one ankle. Suddenly he became sick; the ice-cream was rejected from the stomach; and this was followed by an instantaneous relief of the pain in the foot."

The following case is instructive, impressing us, as it does, with the necessity of scrutinizing all anomalous symptoms that may occur—in other words, of exercising our reasoning faculties in the practice of our profession. It was by a rigid process of induction that the author arrived at the source of the evil in this case.

"A lady consulted me concerning a pain to which she had been for sometime subject, beginning in the left ankle, and extending along the instep towards the little toe, and also into the sole of the foot. The pain was described as being very severe. It was unattended by swelling or redness of the skin, but the foot was tender. She labored also under internal piles, which protruded when she was at the water-closet, at the same time that she lost from them sometimes a large and sometimes a smaller quantity of blood. On a more particular inquiry, I learned that she was free from pain in the foot in the morning; that the pain attacked her as soon as the first evacuation of the bowels had occasioned a protrusion of the piles; that it was especially induced by an evacuation of hard feces; and that if she passed a day without any evacuation at all the pain in the foot never troubled her. Having taken all these facts into consideration, I prescribed her the daily use of a lavement of cold water; that she should take the Ward's paste (*coufectio piperis composita*), three times daily, and some lenitive electuary at bed-time. After having persevered in this plan for the space of six weeks, she called on me again. The piles had now ceased to bleed, and in other respects gave her scarcely any inconvenience. The pain in the foot had entirely left her. She observed that in proportion as the symptoms produced by the piles had abated, the pain in the foot had abated also."

When we first read the lecture on fistula in ano two years ago we were so struck with the views of our author respecting the nature and origin of that disease, that we inserted the lecture in this Journal. It may be seen in the first volume of the new series. The lectures on disease of the hip-joint, that on the use of mercury in syphilis, and several others in this volume, might be profitably copied if we had the space at our disposal. It would not be easy to find in the same compass more useful matter than is embraced in each of these discourses, or indeed in this volume. We the less regret the limited extracts we have it in our power to make from it, because we feel sure that it will in a short time find its way into all the medical libraries in the country.

